# Postmastectomy radiation therapy for autologous breast reconstruction: a systematic review and meta-analysis for the 2022 Japanese Breast Cancer Society Clinical Practice Guideline

**DOI:** 10.1007/s12282-025-01806-3

**Published:** 2025-11-28

**Authors:** Mami Ogita, Subaru Sawayanagi, Haruka Jinnouchi, Michio Yoshimura, Chikako Yamauchi, Naoko Sanuki, Yasushi Hamamoto, Kimiko Hirata, Mariko Kawamura, Yutaka Yamamoto, Shigehira Saji, Tatsuya Toyama

**Affiliations:** 1 Department of Radiology, The University of Tokyo Hospital, 7-3-1 Hongo, Bunkyo- ku, Tokyo, 113-8655 Japan; 2 Department of Radiology, Tokyo Teishin Hospital, 2-14-23 Fujimi, Chiyoda-ku, Tokyo, 102-8798 Japan; 3 Department of Radiation Oncology and Image-Applied Therapy, Graduate School of Medicine, Kyoto University, 54 Shogoin-kawahara-cho, Sakyo-ku, Kyoto, 606-8507 Kyoto Japan; 4 Department of Radiation Oncology, Shiga General Hospital, 5-4-30 Moriyama, Moriyama, 524-8524 Shiga Japan; 5 Department of Radiology, Keio University School of Medicine, 35 Shinanomachi, Shinjuku‑Ku, Tokyo, 160-8582 Japan; 6 Department of Radiation Oncology, National Hospital Organization Shikoku Cancer Center, Ko-160, Minamiumemoto-machi, Matsuyama, Ehime, 791-0280 Japan; 7 Department of Radiation Therapy, Osaka Red Cross Hospital, 5-30 Fudegasakicho, Tennouji-ku, Osaka, 543-8555 Osaka Japan; 8 Department of Radiology, Nagoya University Graduate School of Medicine, 65 Tsurumai-cho, Showa-ku, Nagoya, 466-8550 Aichi Japan; 9 Department of Breast and Endocrine Surgery, Graduate School of Medical Sciences, Kumamoto University, 1-1-1 Honjo, Chuo-ku, Kumamoto, Kumamoto, 860- 8556 Japan; 10 Department of Medical Oncology, Fukushima Medical University, 1 Hikarigaoka, Fukushima, 960-1295 Fukushima Japan; 11 Department of Breast Surgery, Nagoya City University, 1 Kawasumi, Mizuho-cho, Mizuho-ku, Nagoya, 467-8601 Aichi Japan

**Keywords:** Breast cancer, Radiotherapy, Mammaplasty, Reconstruction, Autologous

## Abstract

**Background:**

The safety of postmastectomy radiation therapy (PMRT) after autologous breast reconstruction remains unclear. Therefore, we conducted a systematic review and meta-analysis to investigate the effects of PMRT on patients with breast cancer who underwent autologous breast reconstruction.

**Methods:**

A comprehensive literature search of English and Japanese articles until March 2021 was performed using PubMed/MEDLINE, the Cochrane Library, and Ichushi-Web. We included studies that compared the outcomes of patients with breast cancer who underwent immediate autologous breast reconstruction with and without PMRT. Outcomes including major complications, fat necrosis, and cosmetic results were assessed. Pooled odds ratios (OR) with 95% confidence intervals (CI) were calculated using a random effects model.

**Results:**

Ten studies (two retrospective case-controlled and eight retrospective cohort studies) comprising 3,123 cases were included. The rate of major complications was slightly higher in the PMRT group compared to the no PMRT group, but the difference was not statistically significant (13.2% vs. 12.2%, OR 1.58, 95% CI 0.93–2.68, *P* = 0.09). In contrast, the rate of fat necrosis was significantly increased in the PMRT group (17.2% vs. 8.1%, OR 2.71, 95% CI 1.58–4.65, *P* = 0.0003). Data on cosmetic outcomes were limited and not pooled for the meta-analysis.

**Conclusions:**

PMRT following autologous breast reconstruction was associated with a higher risk of fat necrosis, but not with a significantly increased rate of major complications. With careful patient selection and monitoring, PMRT after autologous breast reconstruction can be considered a safe and acceptable treatment option.

**Supplementary Information:**

The online version contains supplementary material available at 10.1007/s12282-025-01806-3.

## Introduction

Over the years, the number of patients undergoing breast reconstruction has been increasing [1]. Two major methods for breast reconstruction after mastectomy are available: autologous reconstruction, wherein the patient’s own tissue such as skin, fat, and blood vessels are used, and implant-based prosthetic reconstruction, which utilizes silicone implants to recreate the breast shape. Depending on the situation, a combination of implants and autologous flaps can also be used. Autologous breast reconstruction has several advantages over implant-based prosthetic reconstruction. Autologous breasts appear to be more natural and last a lifetime with a single procedure. With no risk of rejection or allergic reactions, concerns regarding implant-related issues are alleviated. Additionally, autologous reconstruction results in higher satisfaction with both the overall outcome and the breast itself and may also reduce the risk of infection and surgical complications [2–4]. The patients chose the reconstruction type based on their health and personal preferences.

A substantial proportion of patients undergoing breast reconstruction after mastectomy require post-mastectomy radiation therapy (PMRT). This therapy has been shown to reduce breast cancer recurrence and mortality in patients with high-risk features, such as positive axillary lymph nodes [5–12]. However, PMRT may be associated with an increased risk of reconstruction-related complications and may negatively affect patient-reported quality of life [13].

Among the various breast reconstruction options available following mastectomy, autologous breast reconstruction is generally considered more resilient to the adverse effects of radiation than implant-based reconstruction. A prospective study showed that autologous reconstruction yielded better patient-reported satisfaction and a lower risk of complications than did implant-based reconstruction in patients undergoing PMRT [14]. Nonetheless, the effect of PMRT on autologous reconstructed breast tissue remains a concern. Several studies have reported that PMRT adversely affects reconstructed breasts. A meta-analysis by Schaverien et al. in 2013 explored this issue and reported no significant differences in the overall complications and revision surgery between patients receiving PMRT and those who did not, although a significantly increased risk of fat necrosis was noted in the PMRT group [15]. Given that the existing evidence is limited and outdated too, particularly with respect to evolving oncoplastic techniques and advancements in radiotherapy, a comprehensive and updated assessment is warranted. Therefore, we conducted an updated systematic review and meta-analysis to evaluate the effect of PMRT on patients undergoing autologous breast reconstruction.

## Materials and methods

The results are reported in accordance with the Preferred Reporting Items for Systematic Reviews and Meta-Analyses (PRISMA) guidelines [16]. The meta-analysis was conducted as a part of the development of the Japanese Breast Cancer Society’s Clinical Practice Guidelines for Radiation Treatment of Breast Cancer, 2022 edition [17].

### Eligibility criteria

We included all randomized or non-randomized controlled trials as well as prospective or retrospective observational studies that evaluated the effect of PMRT on immediate autologous breast reconstruction in breast cancer patients. Articles comparing irradiated and non-irradiated autologous reconstructed breasts groups were included. Review articles, case reports with fewer than 10 patients in one arm, single-arm studies, studies reporting delayed breast reconstruction (breast reconstruction performed after PMRT), and studies that did not assess the outcomes specified for this review were excluded. The studies were limited to those published in English or Japanese.

### Search strategy

The PubMed/MEDLINE, Cochrane Library, and Ichushi-Web databases were systematically searched from January 2016 to March 2021. A combination of MeSH terms and keywords, including “Breast Neoplasms,” “Radiotherapy,” “Mammaplasty,” “Autografts,” “Transplantation, Autologous,” “Breast Implants,” and “Breast Reconstruction,” were used for the literature search (Supplementary Table 1). Additionally, we manually searched the reference lists of the relevant review articles. This review is an updated version of a previous systematic review and meta-analysis conducted in 2018 according to the Japanese Breast Cancer Society’s clinical practice guidelines for breast cancer [9]. The previous systematic review and meta-analysis was performed by searching PubMed/MEDLINE, the Cochrane Library, and Ichushi-Web from the earliest dates until November 2016. Studies selected from the previous meta-analysis were rereviewed and combined in this analysis.

### Selection and data collection process

Two reviewers (SS and HJ) independently reviewed the titles and abstracts from the initial screening. Subsequently, the same two reviewers (SS and HJ) screened the articles by reviewing their full texts. Data were extracted by two independent reviewers (SS and HJ) using a standardized form. Any disagreements were resolved through discussions between the two reviewers (SS and HJ), and if no consensus was reached, a third reviewer (MO) intervened to resolve the issue.

### Outcomes

We assessed major complications, fat necrosis, and cosmesis as the primary outcomes. Major complications were defined as those requiring surgical intervention and/or hospitalization. Any instance of fat necrosis, regardless of its severity, was categorized as ‘fat necrosis’ in this analysis. Regarding cosmesis, outcomes categorized as “good” or “better” were considered acceptable, while those rated below “good” were regarded as a decline in cosmesis.

### Study risk of bias and certainty assessment

 The risk of bias in non-randomized studies was assessed using the Medical Information Distribution Service (Minds) tool [18For each study, two independent reviewers (SS and HJ) evaluated six domains—baseline differences, care differences, outcome measurement, follow-up completeness, confounding adjustment, and other bias—on a scale of high risk (− 2), some concerns (− 1), or low risk (0). Disagreements were resolved by consensus. An overall study-level judgement was assigned according to the Minds manual. A study is classified as “very serious risk” when most domains are scored − 2, as “serious risk” when the domain ratings are mixed (0/−1/−2), and as “no risk” when most domains are scored 0. The levels of indirectness, inconsistency, imprecision, and publication bias were evaluated for each study, and a body of evidence was established for each outcome.

### Data synthesis

We performed a meta-analysis to compare outcomes between patients receiving PMRT to the autologous reconstructed breast and those not receiving PMRT. Odds ratios (OR) and the corresponding 95% confidence intervals (CI) were calculated using random effects models and the inverse-variance method. To account for temporal changes in PMRT techniques, we conducted sensitivity analyses excluding pre-2000 cohorts, using the same random-effects model as the primary analysis. Publication bias was assessed using funnel plots. All tests were two-sided, and a p-value < 0.05 was considered statistically significant. Statistical analysis was performed using Review Manager (RevMan) v5.4 software by the Cochrane Collaboration.

## Results

### Study selection and characteristics

**Fig. 1:**
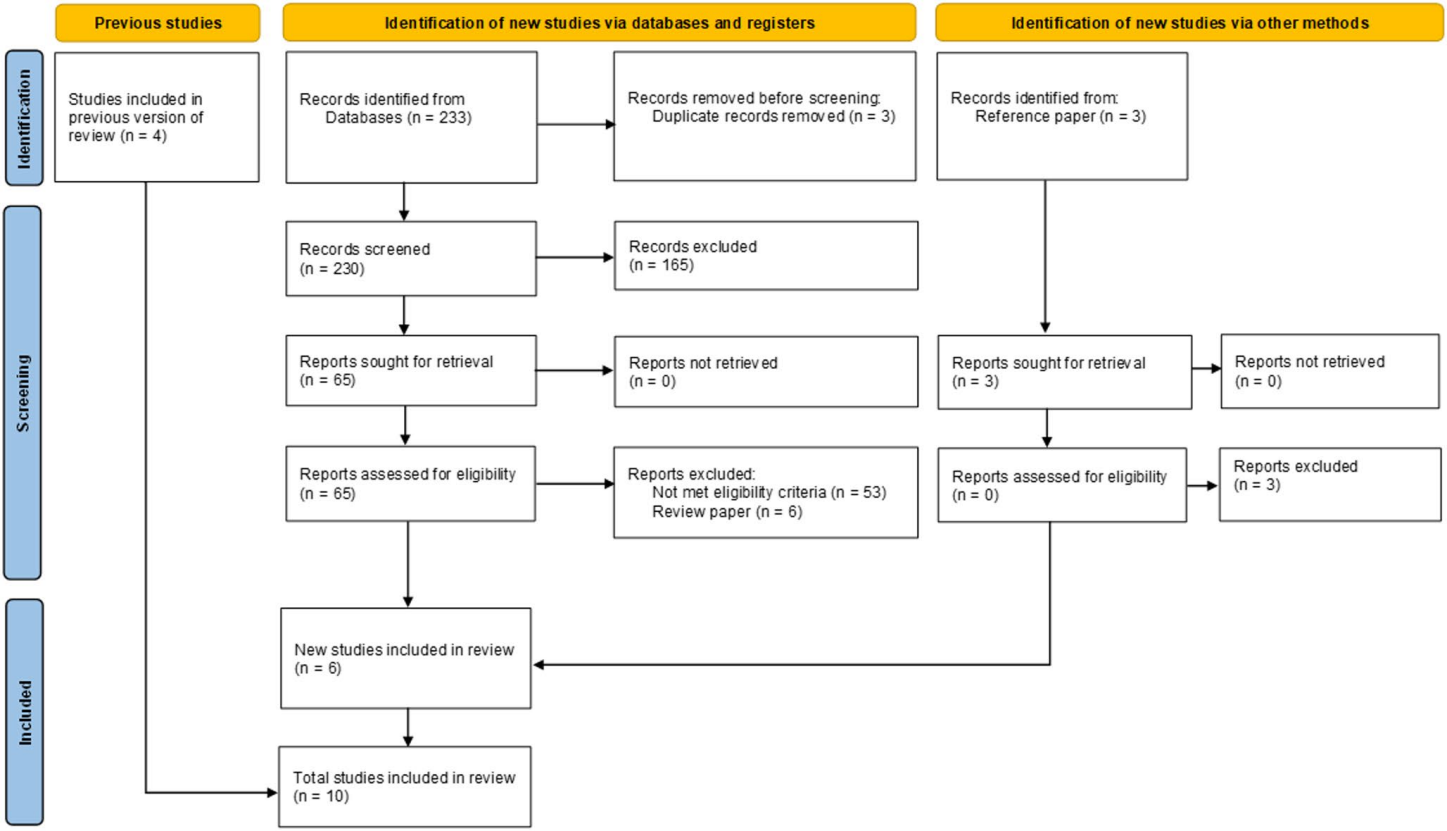
Flow diagram of literature screening according to PRISMA 2020

Our search identified a total of 233 articles. After removing three duplicates, 230 citations remained for screening. We excluded 165 citations after the initial screening and proceeded with the full-text screening of 65 studies. Further, 59 studies were excluded because they did not meet the eligibility criteria. This meta-analysis was conducted with 10 studies, including 6 from the current literature search and 4 from previous analyses. Figure 1 shows the flow diagram of the study selection process. Two retrospective case-control [19, 20] and eight retrospective cohort studies [21–28] were included in this meta-analysis. The study characteristics are summarized in Table 1.

**Table 1:** Characteristics of the included studies

Study	Institution	Study Period	Study Design	Number of Patients or Flaps for Analysis	Follow-up Period (Months)	Type of Autologous Reconstruction	RT Dose to the Reconstructed Breast (Gy)	Boost	Bolus
Tran, NV. 2000	The University of Texas M. D. Anderson Cancer Center, US	1988–1998	Retrospective Cohort	Total 1484 RT 41, No RT 1443	NA	TRAM	Mean 50.99	NA	NA
Rogers, NE. 2002	Memorial Medical Center in New Orleans, US	1994–1999	Case-Control	Total 60 RT 30, No RT 30	Median RT 19.9, No RT 17.4	DIEP	Mean 50.5 (range 44.0-61.2)	NA	NA
Spear, SL. 2005	Georgetown University Hospital, US	1988–1999	Retrospective Cohort	Total 129 RT 38, No RT 91	NA	TRAM	NA	NA	NA
Carlson, GW. 2008	Emory University School of Medicine, US	NA	Retrospective Cohort	Total 174 RT 25, No RT 149	NA	TRAM	NA	NA	NA
Lee, BT. 2010	Guy’s and St Thomas’ NHS Foundation Trust, UK	1999–2006	Retrospective Cohort	Total 407 RT 36, No RT 371	Median RT 63.6, No RT 56.8	LD, TRAM, DIEP, SIEA, SGAP	NA	NA	NA
Taghizadeh, R. 2015	Memorial Sloan Kettering Cancer Center, US	NA	Retrospective Cohort	Total 156 RT 61, No RT 95	Mean RT 33.3, No RT 32.9	DIEP	40–50	Yes (few)	NA
Cooke, AL. 2017	CancerCare Manitoba, Canada	2012–2015	Substudy of Prospective Cohort	Total 125 RT 64, No RT 61	NA	DIEP, SIEA	50-50.4	33%	NA
Myung, Y. 2018	Seoul National University Bundang Hospital, Korea	2012–2016	Retrospective Propensity Score Matched Case-Control Study	Total 42 RT 21, No RT 21	NA	TRAM, DIEP	Mean 52.4	NA	Yes (if necessary)
O’Connell, RL. 2018	The Royal Marsden NHS Foundation Trust, UK	2009–2014	Retrospective Cohort	Total 108 RT 28, No RT 80	Median RT 27.5, No RT 48.7	DIEP	40	NA	Yes (only 1 case)
Zhang, L. 2019	Fudan University Shanghai Cancer Center, China	2001–2015	Retrospective Cohort	Total 438 RT 107, No RT 331	NA	NA	NA	NA	NA

RT: radiation therapy; NA: Not Applicable; TRAM: transverse rectus abdominis musculocutaneous; DIEP: deep inferior epigastric perforators; LD: latissimus dorsi; SIEA: superficial inferior epigastric artery; SGAP: superior gluteal artery perforator

### Risk of bias assessment

Risk of bias was assessed using the Minds approach by two independent reviewers with consensus resolution, rating six domains on a 0/−1/−2 scale. The included studies were not blinded or randomized, as they were observational in nature, and only two studies were adjusted for confounding factors. Using the Minds criteria, most studies were judged to have a serious overall risk of bias, driven mainly by unblinded outcome assessment and heterogeneous adjustment practices,with additional concerns regarding baseline imbalances and care differences. Detailed domain-level ratings for each outcome are provided in Supplementary Tables 2 and 3. Both indirectness and inconsistency were low for all outcomes. A publication bias was noted for fat necrosis (Supplementary Figs. 1 and 2).

### Results of synthesis

#### Major complications

**Fig. 2:**
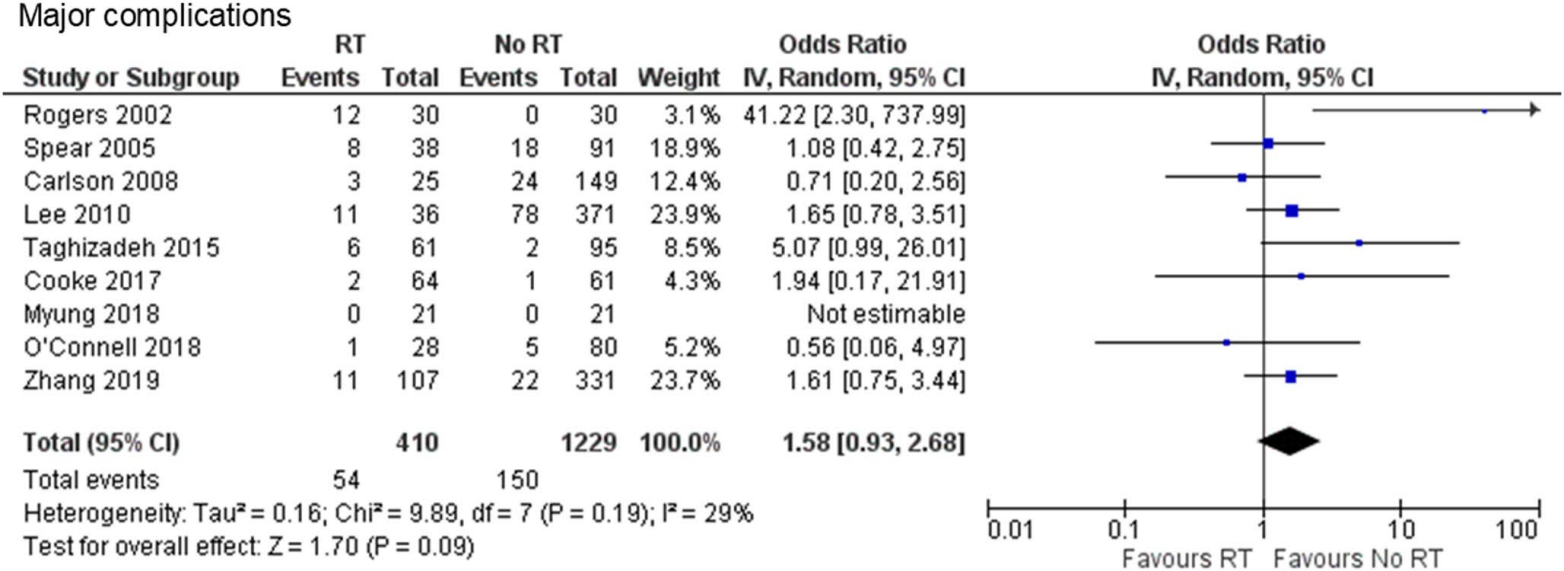
Forest plot illustrating the impact of PMRT on major complications. PMRT: postmastectomy radiation therapy; IV: inverse-variance

Two retrospective case-control [19, 20] and seven retrospective cohort studies [22–28] were included. A total of 1,639 cases were analyzed, including 410 and 1,229 cases in the PMRT and no-PMRT groups, respectively. Major complications were documented to occur in 13.2% of cases in the PMRT group and 12.2% in the no-PMRT group (OR 1.58, 95% CI 0.93–2.68, *P* = 0.09), with no heterogeneity (Chi^2^ = 9.89, df = 7, *P* = 0.19, I^2^ = 29%) (Fig. 2).

### Fat necrosis

**Fig. 3:**
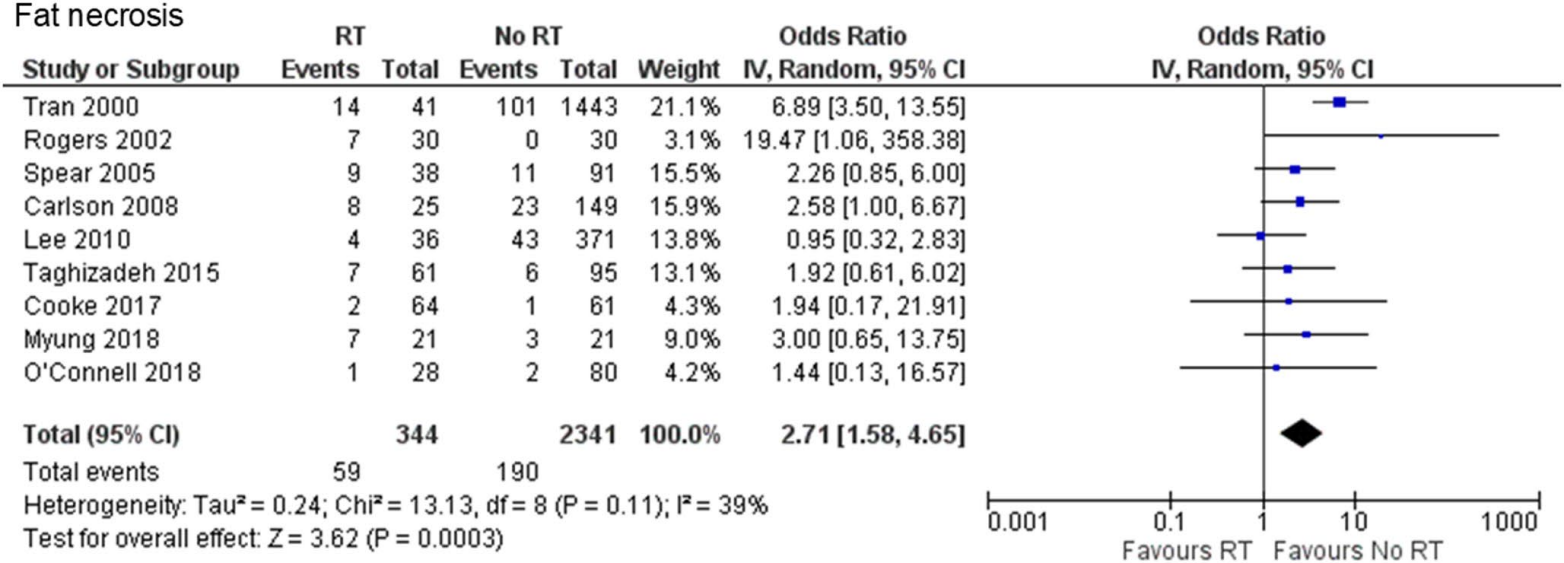
Forest plot illustrating the impact of PMRT on fat necrosis. PMRT: postmastectomy radiation therapy; IV: inverse-variance

Two retrospective case-control [19, 20] and seven retrospective cohort studies [21–27] were included to evaluate fat necrosis. A total of 2,685 cases were analyzed (344 and 2,341 in the PMRT and no-PMRT groups, respectively). Fat necrosis occurred significantly more frequently in the PMRT group than in the no-PMRT group (17.2% vs. 8.1%, OR 2.71, 95% CI 1.58–4.65, *P* = 0.0003), with no heterogeneity (Chi^2^ = 13.13, df = 8, *P* = 0.11, I^2^ = 39%) (Fig. 3).

### Sensitivity analysis

In sensitivity analyses that excluded pre-2000 cohorts, the results remained consistent with the primary analysis, and the conclusions did not change. There was no significant difference in major complications; however, the PMRT group exhibited a higher risk of fat necrosis (Supplementary Fig. 3 and 4). There was no evidence of publication bias for major complications or fat necrosis (Supplementary Fig. 5 and 6).

### Cosmesis

Due to insufficient and heterogeneous reports, a meta-analysis of cosmetic decline could not be performed.

### Certainty of evidence

Supplementary Table 4 presents evidence for each outcome. The certainty of evidence was low for all outcomes.

## Discussion

In this study, we evaluated the effect of PMRT on adverse events in patients with breast cancer who underwent autologous breast reconstruction following mastectomy. Although PMRT was not associated with an increased rate of major complications, it increased the rate of fat necrosis.

Despite a comprehensive literature search, we were unable to find randomized controlled studies of PMRT in patients with breast cancer undergoing autologous breast reconstruction. The indication for PMRT is determined by the disease status, making randomization of the intervention impractical towards clarifing the impact of PMRT on reconstructed breasts. High-quality evidence does not currently exist, and is unlikely to be produced in the future. Available evidence on the effect of PMRT on autologous reconstructed breast tissues is limited. Because no randomized trials exist and the available evidence comes from observational studies, we combined the best available data to estimate pooled effects and their uncertainty. We interpret these findings with caution, acknowledging that residual confounding exists in observational studies. Despite the limited certainty of individual observational studies, a transparently conducted meta-analysis remains useful: it synthesizes evidence from multiple cohorts, clarifies the direction and approximate magnitude of effects, and explicitly reports remaining uncertainties and study limitations.

Among the studies, the rates of major complications varied between 0.0% and 40.0% in the PMRT group and from 0.0% to 21.0% in the no-PMRT group. Most studies included in our analysis did not show statistically significant differences in major complications between the two groups. An exception was the study by Rogers et al. [19], according to which the major complication rate was significantly increased with PMRT (23.3% vs. 0%, OR 41.2, 95% CI 2.3–738.0). However, the study was conducted between 1994 and 1999, and the techniques used for breast reconstruction and radiation therapy are now considered outdated. Therefore, the differences in the complication rates may have been more pronounced.

A previous meta-analysis by Schaverien et al. (2013) demonstrated a significantly increased risk of fat necrosis in patients receiving PMRT compared to those without PMRT (OR 2.8, 95% CI 1.4–5.9, *p* = 0.006), while no significant differences were observed in overall adverse events (OR 1.1, 95% CI 0.8–1.5, *p* = 0.59) or revisional surgery (OR 0.7, 95% CI 0.3–1.7, *p* = 0.38) [15]. Our updated meta-analysis included recently published studies and conducted a formal assessment of the study quality and risk of bias. Our results are consistent with those of Schaverien et al., and confirm a significantly increased incidence of fat necrosis associated with PMRT, while showing no significant increase in major complications. We also conducted sensitivity analyses excluding pre-2000 cohorts to account for differences in PMRT techniques as practice evolved from 2D to CT-based 3D-CRT/IMRT. The pooled estimates and their interpretation were consistent with those of the primary meta-analysis. This supports the robustness of our findings and their relevance to current practice.

 Patient and treatment-related factors likely contribute to the variability in adverse events across studies. Obesity and diabetes are associated with an increased risk of complications in autologous reconstruction [29], while smoking increases wound-related complications, including flap/fat necrosis [30]. Chemotherapy has been associated with increased rates of fat necrosis in some cohorts, but findings are inconsistent [31, 32]. Additionally, reconstructive techniques alter risk profiles: meta-analyses report differences in fat necrosis and related events between pedicled TRAM flaps, free TRAM flaps, and DIEP flaps [33]. Because these variables were inconsistently reported and rarely adjusted for in the included studies, our pooled estimates should be interpreted with caution.

 Although we did not have sufficient data to analyze cosmetic outcomes, several cohort studies suggest that PMRT adversely affects aesthetic appearance after autologous reconstruction. In a propensity-matched series, a decline in cosmesis (fair or poor) was observed in 7 of 21 patients (33.3%) after PMRT, compared to 2 of 21 patients (9.5%) without PMRT. Additionally, flap volume decreased by 12.3% after PMRT, compared to a 2.6% decrease in no PMRT group (*P* < 0.01) [20]. In a cohort using pedicled TRAM flaps with blinded reviewer assessments, PMRT was associated with worse overall aesthetic appearance, symmetry, and greater contracture compared to no PMRT [22]. Blinded photographic evaluations also revealed declines in scores for symmetry, superior-pole contour, and overall proportion in irradiated flaps, while scores improved in non-irradiated controls [19]. A systematic review by Shah et al. concluded that PMRT may worsen cosmetic outcomes in autologous reconstructions, although this has not been definitively proven. Volumetric findings are inconsistent across techniques [34]. In a DIEP-specific cohort, PMRT did not significantly affect flap volume [35]. These data indicate that PMRT may negatively affect cosmesis in at least some autologous reconstructions, though the extent likely varies by flap type, technique, and study populations. To clarify the effect of PMRT, larger studies employing standardized aesthetic endpoints and validated patient-reported outcome measures are warranted.

The patients who underwent immediate implant-based reconstruction with PMRT had a higher risk of reconstruction failure and reported lower satisfaction than those who underwent immediate autologous reconstruction [36]. A recent meta-analysis examining adverse events between patients who underwent immediate implant-based and autologous breast reconstruction in the context of PMRT found that patients who underwent implant-based reconstruction experienced more reconstruction failures than those who underwent autologous reconstruction (RR = 8.61, 95% CI: 2.84–26.08, *P* < 0.05) [37]. Because PMRT for autologous breast reconstruction seems safer than PMRT for prosthetic breast reconstruction, autologous reconstruction may be a more suitable option for patients who require PMRT and wish to undergo breast reconstruction.

This study had several limitations. First, most of the included studies were retrospective in nature, which may carry a risk of selection bias, unmeasured confounding factors, and limitations in data accuracy. Second, differences in reconstructive techniques within autologous breast reconstruction, such as deep inferior epigastric perforator (DIEP), transverse rectus abdominis musculocutaneous (TRAM), and latissimus dorsi flaps, were not analyzed separately. These technique differences may influence the rate of fat necrosis and other complications. Third, the severity of fat necrosis was not distinguished between minor, asymptomatic fat necrosis, and more severe cases requiring intervention, which may have different clinical implications.

In conclusion, although PMRT for autologous breast reconstruction may increase the incidence of fat necrosis, no significant increase in the incidence of major complications was observed. These findings suggest that PMRT is a safe and acceptable treatment option for patients undergoing autologous breast reconstruction. However, given methodological limitations and variability across studies, the applicability and certainty of these conclusions are limited and should be interpreted with caution. Although PMRT should be considered when indicated, clinicians are encouraged to discuss the potential risks of increased complications with patients.

## Supplementary Information

Below is the link to the electronic supplementary material.

Supplementary Material 1
