# Supplementary material for: Postmastectomy radiation therapy for autologous breast reconstruction: a systematic review and meta-analysis for the 2022 Japanese Breast Cancer Society Clinical Practice Guideline
Source: Breast Cancer. 2025 Nov 28;33(1):1–9. doi: 10.1007/s12282-025-01806-3 (PMC12789149; doi:10.1007/s12282-025-01806-3)
Supplement: Supplementary file 1 — Supplementary Material 1 [file 12282_2025_1806_MOESM1_ESM.docx]

**Supplementary Appendix**

Supplementary Table 1. Search strategies on PubMed/MEDLINE

| No | Search Strategy | Result |
| --- | --- | --- |
| #01 | "Breast Neoplasms/radiotherapy"[Mesh] OR ("Breast Neoplasms/therapy"[Mesh] AND "Radiotherapy"[Mesh]) | 20,211 |
| #02 | "Mammaplasty"[Mesh] | 14,682 |
| #03 | "Autografts"[Mesh] OR "Transplantation, Autologous"[Mesh] OR "Breast Implants"[Mesh] | 58,869 |
| #04 | #1 AND #2 AND #3 | 260 |
| #05 | (Breast[TI] OR Mammary[TI]) AND (Tumo*[TI] OR Cancer*[TIAB] OR Carcinoma*[TIAB] OR Neoplasm*[TIAB] OR oncology[TIAB]) AND (mammaplast*[TIAB] OR mammoplast*[TIAB] OR "breast reconstruction"[TIAB]) AND (Radiotherapy[TIAB] OR "radiation therapy"[TIAB] OR irradiation[TIAB]) AND (Autograft*[TIAB] OR Autologous[TIAB] OR implant*[TIAB]) | 386 |
| #06 | #4 OR #5 | 576 |
| #07 | #6 AND 2016/1:2021/3[DP] | 252 |
| #08 | #7 AND (JAPANESE[LA] OR ENGLISH[LA]) | 248 |
| #09 | #8 AND ("Meta-Analysis"[PT] OR "Meta-Analysis as Topic"[Mesh] OR "meta-analysis"[TIAB]) | 14 |
| #10 | #8 AND ("Cochrane Database Syst Rev"[TA] OR "Systematic Review"[PT] OR "Systematic Reviews as Topic"[Mesh] OR "systematic review"[TIAB]) | 13 |
| #11 | #8 AND ("Practice Guideline"[PT] OR "Practice Guidelines as Topic"[Mesh] OR "Consensus"[Mesh] OR "Consensus Development Conferences as Topic"[Mesh] OR "Consensus Development Conference"[PT] OR guideline*[TI] OR consensus[TI]) | 3 |
| #12 | #9 OR #10 OR #11 | 20 |
| #13 | #8 AND ("Randomized Controlled Trial"[PT] OR "Randomized Controlled Trials as Topic"[Mesh] OR (random*[TIAB] NOT medline[SB])) | 6 |
| #14 | #8 AND ("Clinical Trial"[PT] OR "Clinical Trials as Topic"[Mesh] OR "Observational Study"[PT] OR "Observational Studies as Topic"[Mesh] OR (("clinical trial"[TIAB] OR "case control"[TIAB] OR "case comparison"[TIAB]) NOT medline[SB])) | 15 |
| #15 | (#13 OR #14) NOT #12 | 17 |
| #16 | #8 AND ("Epidemiologic Methods"[Mesh] OR "Comparative Study"[PT] OR "Multicenter Study"[PT] OR "Validation Study"[PT] OR ((cohort*[TIAB] OR "comparative study"[TIAB] OR "follow-up"[TIAB] OR "prospective study"[TIAB] OR "Retrospective study"[TIAB]) NOT medline[SB])) | 167 |
| #17 | #16 NOT (#12 OR #15) | 147 |

Supplementary Table 2. Risk of bias assessment for major complications

| Outcomes | | | Major complications requiring a surgical intervention and/or hospitalization | | | | | | | | |  | |  |  | | |  | | |  |  | |  | |  | | |  | | |  |  |  |  |  |  |  |  |  |  |
| --- | --- | --- | --- | --- | --- | --- | --- | --- | --- | --- | --- | --- | --- | --- | --- | --- | --- | --- | --- | --- | --- | --- | --- | --- | --- | --- | --- | --- | --- | --- | --- | --- | --- | --- | --- | --- | --- | --- | --- | --- | --- |
| Individual study | | | Risk of bias | | | | | | | | |  | |  |  | | |  | | |  |  | |  | |  | | |  | | |  |  |  |  |  |  |  |  |  |  |
|  |  |  | Selection bias | Performance bias | | Detection bias | Attrition bias | | Others | | |  | | Factors that can increase the certainty of the evidence | | | | | | |  | Indirectness | | | | | | | | | |  | Number at risk (outcome rate) | | | | | |  |  |  |
| Study code | Study design | | Differences between baseline characteristics | Differences between groups in the care | | Differences between groups in how outcomes are determined | Differences between groups in withdrawals | | Inadequate adjustment for confounding | | Others | Summary | | Dose-response gradient | | | Effect of potential residual confounding factors | | Large magnitude of effect | | Summary | Participants | | | Intervention | | | Comparison | | Outcome | | Summary | The denominator for comparison arm | The numerator for comparison arm | (%) | The denominator for intervention arm | The numerator for intervention arm | (%) | Effect measures | An estimate of effect | 95% Confidence interval |
| Rogers, NE. 2002 | Case-control | | -2 | -1 | | -2 | 0 | | -2 | | 0 | -1 | | 0 | | | 0 | | 0 | | 0 | 0 | | | 0 | | | 0 | | 0 | | 0 | 30 | 0 | 0.0 | 30 | 12 | 40.0 | OR | 41.22 | 2.30–737.99 |
| Spear, SL. 2005 | Retrospective cohort | | -2 | -1 | | -2 | 0 | | -2 | | 0 | -1 | | 0 | | | 0 | | 0 | | 0 | -2 | | | 0 | | | 0 | | 0 | | -1 | 91 | 18 | 19.8 | 38 | 8 | 21.1 | OR | 1.08 | 0.42–2.75 |
| Carlson, GW. 2008 | Retrospective cohort | | -2 | -1 | | -2 | -1 | | -2 | | 0 | -1 | | 0 | | | 0 | | 0 | | 0 | 0 | | | 0 | | | 0 | | 0 | | 0 | 149 | 24 | 16.1 | 25 | 3 | 12.0 | OR | 0.71 | 0.20–2.56 |
| Lee, BT. 2010 | Retrospective cohort | | -2 | -2 | | -2 | 0 | | -2 | | 0 | -2 | | 0 | | | 0 | | 0 | | 0 | -2 | | | 0 | | | 0 | | 0 | | -1 | 371 | 78 | 21.0 | 36 | 11 | 30.6 | OR | 1.65 | 0.78–3.51 |
| Taghizadeh, R. 2015 | Retrospective cohort | | -2 | -1 | | -2 | 0 | | -2 | | 0 | 1 | | 0 | | | 0 | | 0 | | 0 | 0 | | | 0 | | | 0 | | 0 | | 0 | 95 | 2 | 2.1 | 61 | 6 | 9.8 | OR | 5.07 | 0.99–26.01 |
| Cooke, AL. 2017 | Sub of prospective cohort | | -2 | -1 | | -2 | -1 | | -2 | | 0 | 1 | | 0 | | | 0 | | 0 | | 0 | 0 | | | 0 | | | 0 | | 0 | | 0 | 61 | 1 | 1.6 | 64 | 2 | 3.1 | OR | 1.94 | 0.17–21.91 |
| Myung, Y. 2018 | Retrospective propensity score matched case-control | | -2 | -1 | | -2 | -1 | | 0 | | 0 | -1 | | 0 | | | 0 | | 0 | | 0 | 0 | | | 0 | | | 0 | | 0 | | 0 | 21 | 0 | 0.0 | 21 | 0 | 0.0 | OR | NA | NA |
| O’Connell, RL. 2018 | Retrospective cohort | | -2 | -1 | | -2 | 0 | | -2 | | 0 | -1 | | 0 | | | 0 | | 0 | | 0 | 0 | | | 0 | | | 0 | | 0 | | 0 | 80 | 5 | 6.3 | 28 | 1 | 3.6 | OR | 0.56 | 0.06–4.97 |
| Zhang, L. 2019 | Retrospective cohort | | -2 | -2 | | -2 | 0 | | -1 | | 0 | -1 | | 0 | | | 0 | | 0 | | 0 | -2 | | | 0 | | | 0 | | -2 | | -1 | 331 | 22 | 6.6 | 107 | 11 | 10.3 | OR | 1.61 | 0.75–3.44 |
| Comment |  |  | | |  | | |  | |  | | |  | | |  | | | |  | | |  | | | |  | | | |  | |  |  |  |  |  |  |  |  |  |
| Rogers, NE. 2002 |  | | TNM status unknown. | The details of chemotherapy and endocrine therapy were not specified. | | Unblinded |  | | No adjustment for confounding was performed. | |  |  | |  | | |  | |  | |  |  | | |  | | |  | |  | |  |  |  |  |  |  |  |  |  |  |
| Spear, SL. 2005 |  | | TNM status unknown. | The details of chemotherapy and endocrine therapy were not specified. | | Unblinded |  | | No adjustment for confounding was performed. | |  |  | |  | | |  | |  | |  | Immediate/delayed reconstruction was mixed. | | |  | | |  | |  | |  |  |  |  |  |  |  |  |  |  |
| Carlson, GW. 2008 |  | | TNM status unknown. | The details of chemotherapy and endocrine therapy were not specified. | | Unblinded | Follow-up duration unknown | | No adjustment for confounding was performed. | |  |  | |  | | |  | |  | |  |  | | |  | | |  | |  | |  |  |  |  |  |  |  |  |  |  |
| Lee, BT. 2010 |  | | TNM status unknown. Difference in reconstruction timing. | The details of chemotherapy and endocrine therapy were not specified. | | Unblinded |  | | No adjustment for confounding was performed. | |  |  | |  | | |  | |  | |  | Immediate/delayed reconstruction was mixed. | | |  | | |  | |  | |  |  |  |  |  |  |  |  |  |  |
| Taghizadeh, R. 2015 |  | | Difference in TNM status and chemotherapy. | The details of chemotherapy and endocrine therapy were not specified. | | Unblinded |  | | No adjustment for confounding was performed. | |  |  | |  | | |  | |  | |  |  | | |  | | |  | |  | |  |  |  |  |  |  |  |  |  |  |
| Cooke, AL. 2017 |  | | Difference in TNM status. | The details of chemotherapy and endocrine therapy were not specified. | | Unblinded | Six cases were excluded because of breast cancer death within one year. | | No adjustment for confounding was performed. | |  |  | |  | | |  | |  | |  |  | | |  | | |  | |  | |  |  |  |  |  |  |  |  |  |  |
| Myung, Y. 2018 |  | | TNM status unknown. | The details of chemotherapy and endocrine therapy were not specified. | | Unblinded | Follow-up duration unknown | |  | |  |  | |  | | |  | |  | |  |  | | |  | | |  | |  | |  |  |  |  |  |  |  |  |  |  |
| O’Connell, RL. 2018 |  | | Difference in TNM status and chemotherapy. | The details of chemotherapy and endocrine therapy were not specified. | | Unblinded |  | | No adjustment for confounding was performed. | |  |  | |  | | |  | |  | |  |  | | |  | | |  | |  | |  |  |  |  |  |  |  |  |  |  |
| Zhang, L. 2019 |  | | TNM status unknown. | Difference in chemotherapy. | | Unblinded |  | | No TNM adjustment was performed in the multivariate analysis. | |  |  | |  | | |  | |  | |  | Some delayed reconstruction was included. | | |  | | |  | | Re-operation for cosmesis was included. | |  |  |  |  |  |  |  |  |  |  |

Supplementary Table 3. Risk of bias assessment for fat necrosis

| Outcomes | | Fat necrosis | | | | | | | | | |  |  |  |  |  |  | |  |  | |  | |  |  |  |  |  |  |  |  |  |  |
| --- | --- | --- | --- | --- | --- | --- | --- | --- | --- | --- | --- | --- | --- | --- | --- | --- | --- | --- | --- | --- | --- | --- | --- | --- | --- | --- | --- | --- | --- | --- | --- | --- | --- |
| Individual study | | Risk of bias | | | | | | | | | |  |  |  |  |  |  | |  |  | |  | |  |  |  |  |  |  |  |  |  |  |
|  |  | Selection bias | Performance bias | | | Detection bias | Attrition bias | | Others | | |  | Factors that can increase the certainty of the evidence | | |  | Indirectness | | | | | | |  | Number at risk (outcome rate) | | | | | |  |  |  |
| Study code | Study design | Differences between baseline characteristics | Differences between groups in the care | | Differences between groups in how outcomes are determined | | | Differences between groups in withdrawals | | Inadequate adjustment for confounding | Others | Summary | Dose-response gradient | Effect of potential residual confounding factors | Large magnitude of effect | Summary | Participants | Intervention | | | Comparison | | Outcome | Summary | The denominator for comparison arm | The numerator for comparison arm | (%) | The denominator for intervention arm | The numerator for intervention arm | (%) | Effect measures | An estimate of effect | 95% Confidence interval |
| Tran, NV. 2000 | Retrospective cohort | -2 | -1 | | -2 | | | 0 | | -2 | 0 | -1 | 0 | 0 | 0 | 0 | 0 | 0 | | | 0 | | 0 | 0 | 1443 | 101 | 7.0 | 41 | 14 | 34.1 | OR | 6.89 | 3.50–13.55 |
| Rogers, NE. 2002 | Case-control | -2 | -1 | | -2 | | | 0 | | -2 | 0 | -1 | 0 | 0 | 0 | 0 | 0 | 0 | | | 0 | | 0 | 0 | 30 | 0 | 0.0 | 30 | 7 | 23.3 | OR | 19.47 | 1.06–358.38 |
| Spear, SL. 2005 | Retrospective cohort | -2 | -1 | | -2 | | | 0 | | -2 | 0 | -1 | 0 | 0 | 0 | 0 | -2 | 0 | | | 0 | | 0 | -1 | 91 | 11 | 12.1 | 38 | 9 | 23.7 | OR | 2.26 | 0.85–6.00 |
| Carlson, GW. 2008 | Retrospective cohort | -2 | -1 | | -2 | | | -1 | | -2 | 0 | -1 | 0 | 0 | 0 | 0 | 0 | 0 | | | 0 | | 0 | 0 | 149 | 23 | 15.4 | 25 | 8 | 32.0 | OR | 2.58 | 1.00–6.67 |
| Lee, BT. 2010 | Retrospective cohort | -2 | -2 | | -2 | | | 0 | | -2 | 0 | -2 | 0 | 0 | 0 | 0 | -2 | 0 | | | 0 | | 0 | -1 | 371 | 43 | 11.6 | 36 | 4 | 11.1 | OR | 0.95 | 0.32–2.83 |
| Taghizadeh, R. 2015 | Retrospective cohort | -2 | -1 | | -2 | | | 0 | | -2 | 0 | 1 | 0 | 0 | 0 | 0 | 0 | 0 | | | 0 | | 0 | 0 | 95 | 6 | 6.3 | 61 | 7 | 11.5 | OR | 1.92 | 0.61–6.02 |
| Cooke, AL. 2017 | Sub of prospective cohort | -2 | -1 | | -2 | | | -1 | | -2 | 0 | 1 | 0 | 0 | 0 | 0 | 0 | 0 | | | 0 | | 0 | 0 | 61 | 1 | 1.6 | 64 | 2 | 3.1 | OR | 1.94 | 0.17–21.91 |
| Myung, Y. 2018 | Retrospective propensity score matched case-control | -2 | -1 | | -2 | | | -1 | | 0 | 0 | -1 | 0 | 0 | 0 | 0 | 0 | 0 | | | 0 | | 0 | 0 | 21 | 3 | 14.3 | 21 | 7 | 33.3 | OR | 3 | 0.65–13.75 |
| O’Connell, RL. 2018 | Retrospective cohort | -2 | -1 | | -2 | | | 0 | | -2 | 0 | -1 | 0 | 0 | 0 | 0 | 0 | 0 | | | 0 | | 0 | 0 | 80 | 2 | 2.5 | 28 | 1 | 3.6 | OR | 1.44 | 0.13–16.57 |
| Comment | | | |  | | | | | | | |  |  |  |  |  |  |  | | |  | |  |  |  |  |  |  |  |  |  |  |  |
| Tran, NV. 2000 |  | Difference in TNM status. | The details of chemotherapy and endocrine therapy were not specified. | | Unblinded | | | The details of follow-up duration were not specified. | | No adjustment for confounding was performed. |  |  |  |  |  |  |  |  | | |  | |  |  |  |  |  |  |  |  |  |  |  |
| Rogers, NE. 2002 |  | TNM status unknown. | The details of chemotherapy and endocrine therapy were not specified. | | Unblinded | | |  | | No adjustment for confounding was performed. |  |  |  |  |  |  |  |  | | |  | |  |  |  |  |  |  |  |  |  |  |  |
| Spear, SL. 2005 |  | TNM status unknown. | The details of chemotherapy and endocrine therapy were not specified. | | Unblinded | | |  | | No adjustment for confounding was performed. |  |  |  |  |  |  | Immediate/delayed reconstruction was mixed. |  | | |  | |  |  |  |  |  |  |  |  |  |  |  |
| Carlson, GW. 2008 |  | TNM status unknown. | The details of chemotherapy and endocrine therapy were not specified. | | Unblinded | | | Follow-up duration unknown | | No adjustment for confounding was performed. |  |  |  |  |  |  |  |  | | |  | |  |  |  |  |  |  |  |  |  |  |  |
| Lee, BT. 2010 |  | TNM status and reconstruction timing were unknown. | The details of chemotherapy and endocrine therapy were not specified. | | Unblinded | | |  | | No adjustment for confounding was performed. |  |  |  |  |  |  | Immediate/delayed reconstruction was mixed. |  | | |  | |  |  |  |  |  |  |  |  |  |  |  |
| Taghizadeh, R. 2015 |  | Difference in TNM status and chemotherapy. | The details of chemotherapy and endocrine therapy were not specified. | | Unblinded | | |  | | No adjustment for confounding was performed. |  |  |  |  |  |  |  |  | | |  | |  |  |  |  |  |  |  |  |  |  |  |
| Cooke, AL. 2017 |  | Difference in TNM status. | The details of chemotherapy and endocrine therapy were not specified. | | Unblinded | | | Six cases were excluded because of breast cancer death within one year. | | No adjustment for confounding was performed. |  |  |  |  |  |  |  |  | | |  | |  |  |  |  |  |  |  |  |  |  |  |
| Myung, Y. 2018 |  | TNM status unknown. | The details of chemotherapy and endocrine therapy were not specified. | | Unblinded | | | Follow-up duration unknown | |  |  |  |  |  |  |  |  |  | | |  | |  |  |  |  |  |  |  |  |  |  |  |
| O’Connell, RL. 2018 |  | Difference in TNM status and chemotherapy and endocrine therapy. | The details of chemotherapy and endocrine therapy were not specified. | | Unblinded | | |  | | No adjustment for confounding was performed. |  |  |  |  |  |  |  |  | | |  | |  |  |  |  |  |  |  |  |  |  |  |

Supplementary Table 4. Body of evidence by each outcome

|  |  |  |  |  |  |  |  | Number at risk (outcome rate) | | | | | |  |  |  |  |  |
| --- | --- | --- | --- | --- | --- | --- | --- | --- | --- | --- | --- | --- | --- | --- | --- | --- | --- | --- |
| Outcomes | Study design /Number of studies | Risk of bias | Inconsistency | Imprecision | Indirectness | Others (Publication bias) | Factors that can increase the certainty of the evidence | The denominator for comparison arm | The numerator for comparison arm | (％) | The denominator for intervention arm | The numerator for intervention arm | (％) | Effect measures | An estimate of effect | 95% Confidence interval | Certainty of evidence | Importance |
| Major complications | Cohort /7, Case-control /2 | -1 | 0 | 0 | 0 | 0 | 0 | 1229 | 150 | 12.2 | 410 | 54 | 13.2 | OR | 1.58 | 0.93–2.68 | Low (C) | 8 |
| Fat necrosis | Cohort /7, Case-control /2 | -1 | 0 | 0 | 0 | -1 | 0 | 2341 | 190 | 8.1 | 344 | 59 | 17.2 | OR | 2.71 | 1.58–4.65 | Low (C) | 8 |


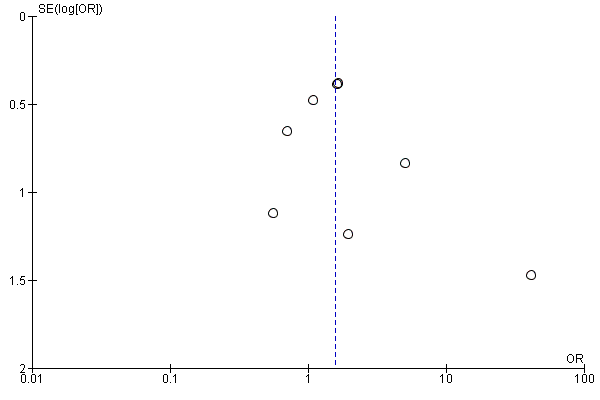


Supplementary Figure 1. Funnel plot evaluating publication bias for major complications


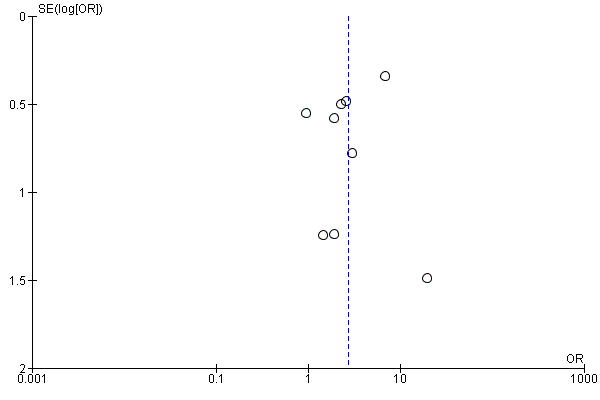


Supplementary Figure 2. Funnel plot evaluating publication bias for fat necrosis


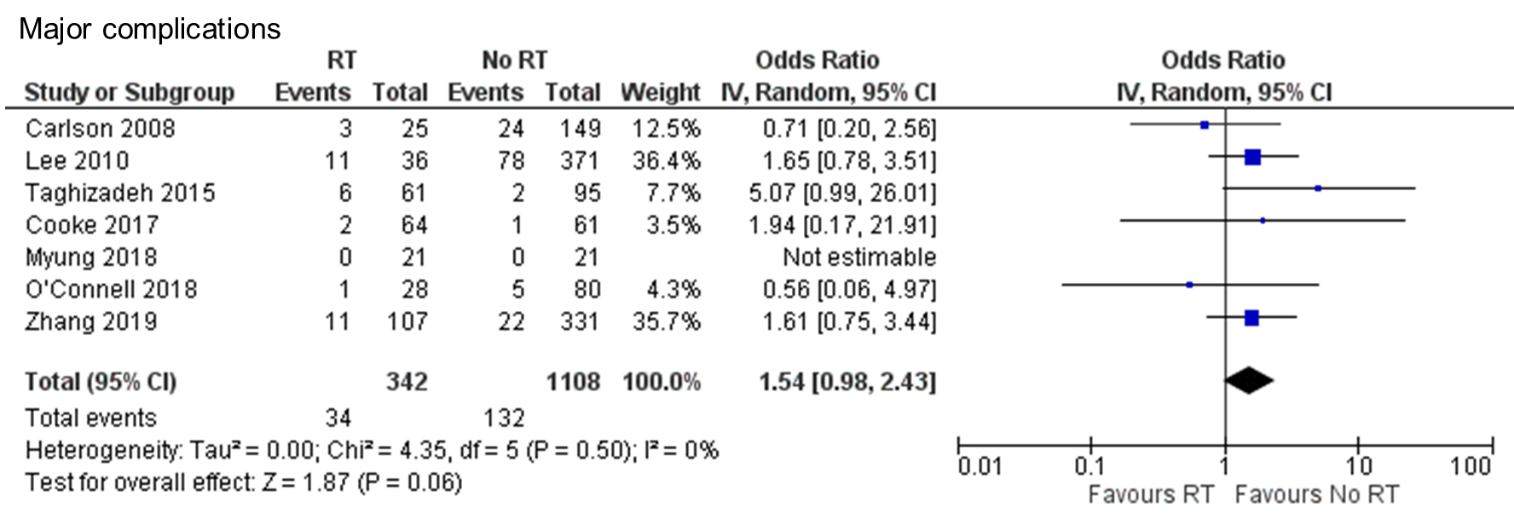


Supplementary Figure 3. Forest plot illustrating the impact of PMRT on major complications: sensitivity analysis excluding pre-2000 cohorts

PMRT: postmastectomy radiation therapy; IV: inverse-variance


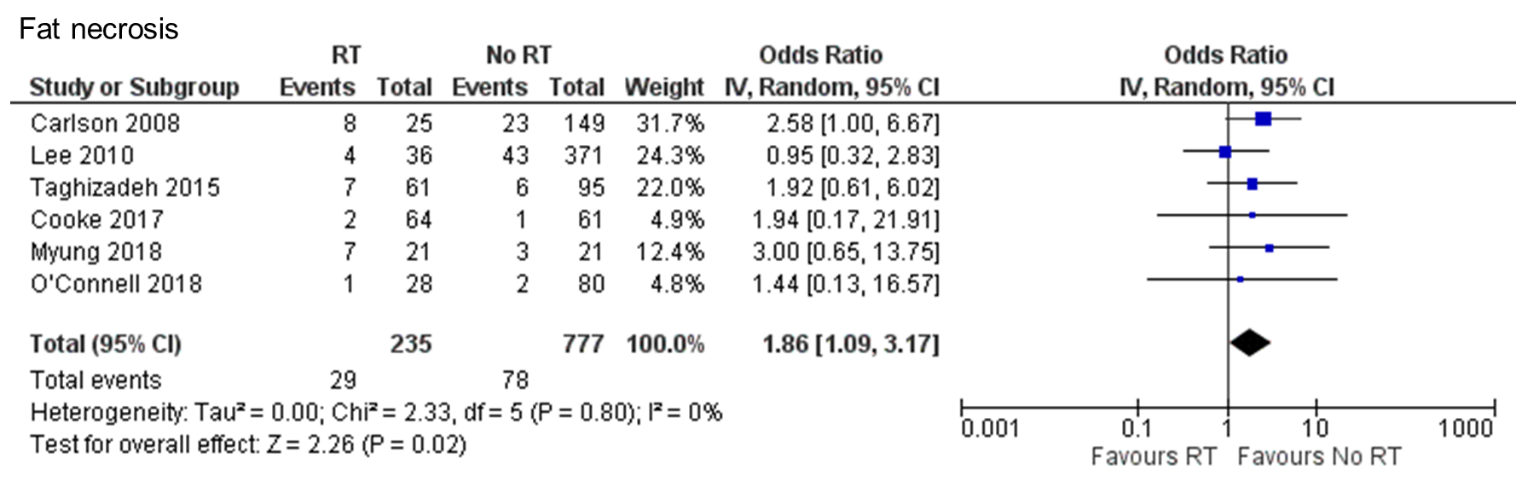


Supplementary Figure 4. Forest plot illustrating the impact of PMRT on fat necrosis: sensitivity analysis excluding pre-2000 cohorts

PMRT: postmastectomy radiation therapy; IV: inverse-variance


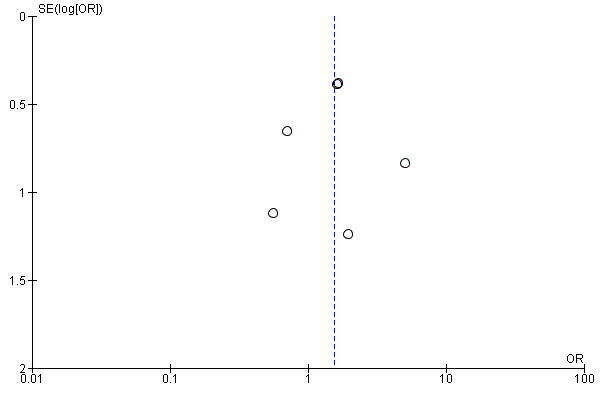


Supplementary Figure 5. Funnel plot evaluating publication bias for major complications: sensitivity analysis excluding pre-2000 cohorts


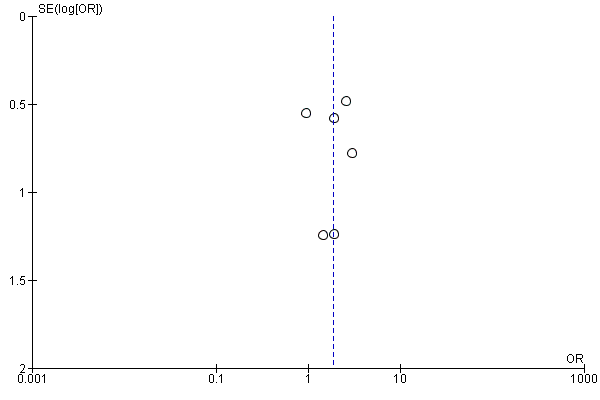


Supplementary Figure 6. Funnel plot evaluating publication bias for fat necrosis: sensitivity analysis excluding pre-2000 cohorts
